# London Hospital

**Published:** 1830-10

**Authors:** 


### LONDON HOSPITAL.

### *Efficacy of Croton Oil in Cases of obstinate Constipation, &c.**

Case I. John Hickman, ætatis twenty-one, was admitted an in-patient of the hospital on the 24th of August. He represented his illness to have commenced ten days before, with a violent twisting pain in the bowels, attended by constipation. During that time his medical attendant had given him 160 grains of calomel, a pound and a half of salts, and a pound of castor oil, besides venesection, twice, to the amount of sixteen ounces each time, with enemata innumerable. The removal of this patient from his bed to the hospital caused a general depression. On his arrival, his pulse was scarcely perceptible: pain on pressure of the abdomen. Two pills, each containing one minim of Croton Oil, were given immediately, and, as re-action did not take place for some time, he was put into a warm bath. When put to bed a second time, the bowels began to act, and continued all night and part of the following day, when an immense quantity of feculent matter was dislodged: by this all pain and uneasiness was removed, and in a fortnight he was discharged, cured.

Case II. Mary Ann Robertson, ætatis sixteen, reported on her admission that, for four months before, her belly had gradually increased in size; that she had been under medical treatment out of doors, but without having derived any benefit.

After her admission, three days were allowed to pass without entering on any particular plan of treatment, as the fluctuation was very obscure. After the lapse of that time, two minims of Croton Oil were administered in the evening, which began to operate about twelve o'clock, P.M. The number of stools were not counted by this patient, as she was greatly distressed by the involuntary discharge of urine. So great was the quantity discharged, that it ran through the bed, and literally overflowed the ward: the tumefaction entirely subsided; and in ten days she was discharged cured.

* From Dr. Short's Practical Remarks on Croton Oil.

